# Stimulation of Apolipoprotein A-IV expression in Caco-2/TC7 enterocytes and reduction of triglyceride formation in 3T3-L1 adipocytes by potential anti-obesity Chinese herbal medicines

**DOI:** 10.1186/1749-8546-4-5

**Published:** 2009-03-26

**Authors:** Ava Jiangyang Guo, Roy Chi-yan Choi, Anna Wing-han Cheung, Jun Li, Ivy Xiaoying Chen, Tina Tingxia Dong, Karl Wah-keung Tsim, Brad Wing-chuen Lau

**Affiliations:** 1 Department of Biology and the Center for Chinese Medicine, Hong Kong University of Science and Technology, Clear Water Bay Road, Hong Kong SAR, PR China; 2 Macao Institute for Applied Research in Medicine and Health (MUST Foundation), Avenida Wai Long, Taipa, Macao SAR, PR China

## Abstract

**Background:**

Chinese medicine has been proposed as a novel strategy for the prevention of metabolic disorders such as obesity. The present study tested 17 Chinese medicinal herbs were tested for their potential anti-obesity effects.

**Methods:**

The herbs were evaluated in terms of their abilities to stimulate the transcription of Apolipoprotein A-IV (ApoA-IV) in cultured Caco-2/TC7 enterocytes. The herbs that showed stimulating effects on ApoA-IV transcription were further evaluated in terms of their abilities to reduce the formation of triglyceride in differentiated 3T3-L1 adipocytes.

**Results:**

ApoA-IV transcription was stimulated by *Rhizoma Alismatis *and *Radix Angelica Sinensis *in a dose- and time-dependent manner in cultured Caco-2/TC7 cells. Moreover, these two herbs reduced the amount of triglyceride in differentiated 3T3-L1 adipocytes.

**Conclusion:**

The results suggest that *Rhizoma Alistmatis *and *Radix Angelica Sinensis *may have potential anti-obesity effects as they stimulate ApoA-IV transcription and reduce triglyceride formation.

## Background

Obesity is one of the metabolic disorders attributed to various factors such as uncontrolled food intake, environment and lack of exercises. Excessive weight may be a precursor of serious illnesses including diabetes, heart disease and cancer [1]. More and more people in China now live a sedentary lifestyle and consume calorie-rich foods [2]. Between 1992 and 2002, more than 60 million people became obese in China [3] where the prevalence of obesity is likely to increase [4-6]. By 2020, the obese population in China is expected to surpass that in the United States [7].

The current choices for anti-obesity medications are quite limited and some anti-obesity medicines have serious or even life-threatening side effects [8]. There is a pressing need for new and/or alternative treatments against obesity.

Chinese medicine was found useful in preventing and treating obesity. For example, tea polyphenols, especially epigallocatechin-3-gallate (EGCG), which increases apoptosis in mature adipocyte, was proposed to be a chemo-preventive agent against obesity [9]. Ginsenoside Rh2 may prevent obesity via the AMPK signaling pathway [10].

Apolipoprotein A-IV (ApoA-IV), a circulating glycoprotein primarily synthesized in the small intestines during fat absorption [11], was demonstrated to prevent atherosclerosis by modulating plasma lipoprotein metabolism [12] and inhibit gastric motility, acid secretion [13-15] and intestinal motility [16]. More importantly, ApoA-IV may be involved in the control of food intake [17-19]. Ingestion of food containing high lipid content produces chylomicrons, which are absorbed by intestinal cells to trigger the synthesis and secretion of ApoA-IV into blood [20]. ApoA-IV is also synthesised and regulated in the hypothalamus [21]. Recently, Gotoh *et al. *suggested that the action of ApoA-IV took place in our central nervous system; high levels of ApoA-IV in blood reduce food intake by potentiating the anorectic effect of central melanocortin agonists [19]. Hypothalamic melanocortin system is critical in the regulation of food intake and body weight [22]. ApoA-IV gene regulation may serve as a negative feedback circuit to control food intake.

Adipogenesis is another potential target for treating obesity. Several cell types were shown to undergo *in vitro *lipogenic differentiation into adipocytes, including the well characterized 3T3-L1 pre-adipocytes [23-26]. Induced by a chemical cocktail, 3T3-L1 cells differentiate to form adipocytes, with the accumulation of triglyceride (TG) as one of the hallmarks of adipogenesis. The anti-obesity effect therefore could be represented by the suppression of TG formation in 3T3-L1 adipocytes.

In the present study, 17 Chinese medicinal herbs were evaluated for their potential anti-obesity effects in terms of their abilities to stimulate ApoA-IV expression and TG formation.

To demonstrate the potential anti-obesity effects of the Chinese medicinal herbs, we employ an intestinal cell line Caco-2/TC7 stably transfected by a human ApoA-IV promoter tagged with a firefly luciferase gene [27]. The high sensitivity in the measurement of luciferase allows us to evaluate the transcriptional activation or repression of the ApoA-IV promoter by the herbs. Those herbs with significant effects on ApoA-IV transcription were further analyzed in terms of the TG content in differentiated 3T3-L1 adipocytes.

## Methods

### Raw materials

The Chinese medicinal herbs in this study were A: *Rhizoma Alismatis *(*Zexie*), B: *Fructus Crataegi *(*Shanzha*), C: *Semen Coicis *(*Yiyiren*), D: *Rhizoma Atractylodis Macrocephalae *(*Baizhu*), E:*Rhizoma Atractylodis *(*Cangzhu*), F:*Sclerotium Poriae Cocos *(*Fuling*), G: *Semen Cassiae *(*Juemingzi*), H: *Folium Sennae *(*Fanxieye*), I: *Radix Angelica Sinensis *(*Danggui*), J: *Rhizoma Curumae *(*Ezhu*), K: *Flos Chrysanthemi *(*Juhua*), L: *Radix Notoginseng *(*Sanqi*), M: *Folium Nelumbinis *(*Heye*), N: *Herba Taraxaci *(*Pugongying*), O: *Pericarpium Citri Reticulatae *(*Chenpi*), P: *Fructus Schisandrae Chinensis *(*Wuweizi*) and Q: *Fructus Mori *(*Sangshen*). All the herbs were purchased from Eu Yan Sang International Ltd and Tung Fong Hong Medicine Co Ltd in Hong Kong and were authenticated by organoleptic characteristics according to the Pharmacopoeia of the People's Republic of China (2005 edition, volume I). Each experimental species (300 g) was deposited in the Herbarium of the Department of Biology, Hong Kong University of Science and Technology (Additional file 1).

These herbs were divided into two groups. The first group includes the herbs that treat obesity or obesity-related diseases according to the Pharmacopoeia of the People's Republic of China and other literature. The second group includes common dietary herbs not documented to have functional effects on obesity.

### Preparation of herbal extracts

Two methods, namely water and ethanol extractions, were used to prepare herbal extracts in the present study. In water extraction, each herb was ground and boiled twice in eight units of water for one hour. In ethanol extraction, each grounded herb was immersed in eight units of 95% ethanol for two hours and reflux for further two hours. Both water and ethanol extracts were dried into powder and stored at -80°C.

### Cell culture

The stable cell line of Caco-2/TC7 transfected with the human ApoA-IV promoter was provided by Prof M Lacasa (Université Pierre et Marie Curie, France). TC7 is the selected clone from Caco-2 cells [28]. The cells were grown at 37°C in a water-saturated incubator containing 5% CO_2 _in Dulbecco's Modified Eagle's Medium (DMEM) supplemented with 20% heat-inactivated fetal bovine serum (HI-FBS), 100 U/ml penicillin and 100 μg/ml streptomycin. In all experiments, cells were maintained at about 90% confluence. The high confluence condition allowed cell differentiation, which increases the expression of ApoA-IV. Prior to drug treatment, Caco-2/TC7 cells were seeded on 24-well microtiter plates (40000 cells/well) for 24 hours. Mouse 3T3-L1 fibroblast cells (ATCC no.CL-173) were obtained from American Type Culture Collection (USA) and maintained at 37°C in a water-saturated incubator containing 5% CO_2 _and in DMEM supplemented with 4.5 g/L glucose, 10% FBS, 100 U/ml penicillin and 100 μg/ml streptomycin. Induction of lipogenic differentiation was detailed in a previous study [29]. Briefly, the confluent cultures were treated with a differentiation cocktail containing dexamethasone (1 μM, Sigma, USA), insulin (1.8 μM, Sigma, USA) and dibutryl-cAMP (300 μM, Sigma, USA) for 72 hours to induce lipogenesis. The cultures were set as day 0, and replaced with the culture medium containing insulin (1.8 μM) for every two days. At day 10, about 80% of cultures were induced to contain triglyceride (TG). Treatments including serum starvation (DMEM only), insulin (1.8 μM), *Radix Angelica Sinensis *and *Rhizoma Alistmatis *(10, 1 and 0.1 mg/ml water extracts) were given to differentiated cultures (on day 10) for 72 hours. Unless described otherwise, all the culture reagents were purchased from Invitrogen Technologies (USA).

### Preparation of lipid micelles

The lipid micelles was used to mimic the duodenal micelles resulting from digestion of lipid [30], and prepared according to the method of Carrière *et al. *[27]. The stock solution contained 0.6 mM oleic acid, 0.2 mM L-α-lysophosphatidylcholine, 0.05 mM cholesterol, 0.2 mM 2-monooleoylglycerol and 2 mM taurocholic acid, and used in the dilutions from 1:1000 to 1:3000.

### Luciferase assay

Luciferase assay was performed with a commercial kit (Tropix, USA). Briefly, the treated Caco-2/TC7 cells were collected and re-suspended by 0.2% Triton X-100, 1 mM dithiothreitol and 100 mM potassium phosphate buffer (pH7.8). The lysate was subjected to luciferase assay and protein assay. The luminescent reaction was measured by Tropix TR717 microplate luminometer, while the protein concentrations were measured according to the Bradford method [31] with a protein assay kit (Bio-Rad Laboratories, USA). The luciferase activity reading was normalized by protein amount in the sample.

### Quantitative PCR analysis

Total RNAs, isolated by TRIzol reagent (Invitrogen, USA) from treated Caco-2/TC7 cultures, were reverse-transcribed to cDNAs by Moloney murine leukemia virus reverse transcriptase (Invitrogen, USA) according to the manufacturer's instructions. Quantitative PCR was performed with SYBR Green Master mix and Rox reference dye according to the manufacturer's instructions (Applied Biosystems, USA). The primers used for human ApoA-IV (NM_000482) were 5'-ATG TTC CTG AAG GCC GTG G-3' and 5'-TGC AGG TCA CCT GCG TAA G-3' (-105 to -334), and human18S rRNA (NR_003286) 5'-TGT GAT GCC CTT AGA TGT CC-3' and 5'-GAT AGT CAA GTT CGA CCG TC-3'(-1494 to -1813). The SYBR green signal was detected by a quantitative PCR (Mx3000p multiplex, Stratagene, USA). The relative transcript expression levels were quantified according to the ΔΔCt (cycle threshold) method [32]. The calculation was done with the Ct value of 18S rRNA to normalize the Ct value of target gene in each sample to obtain the ΔCt value, which was then used to compare different samples. The PCR products were analyzed by gel electrophoresis, while the specificity of amplification was confirmed by melting curve.

### Oil red O staining assay

Oil Red O at 0.2% in isopropanol was mixed with water (3:2, v/v) and filtered. Experimental cultured cells were washed with PBS, fixed by paraformaldehyde (4% in PBS, Sigma, USA) for 5 minutes, incubated with filtered Oil Red O for 30 minutes, and washed twice with PBS. The stained TG was extracted by isopropanol and its quantity was measured at 490 nm absorbance [28].

### Statistical Analysis

One-way analysis of variance (ANOVA) was carried out with SPSS software (version 13.0, SPSS, USA). The levels of statistical significance were *P *< 0.05 (*), *P *< 0.01 (**) and *P *< 0.001 (***).

## Results

### Transcriptional activation of ApoA-IV in Caco-2/TC7

**Figure 1:**
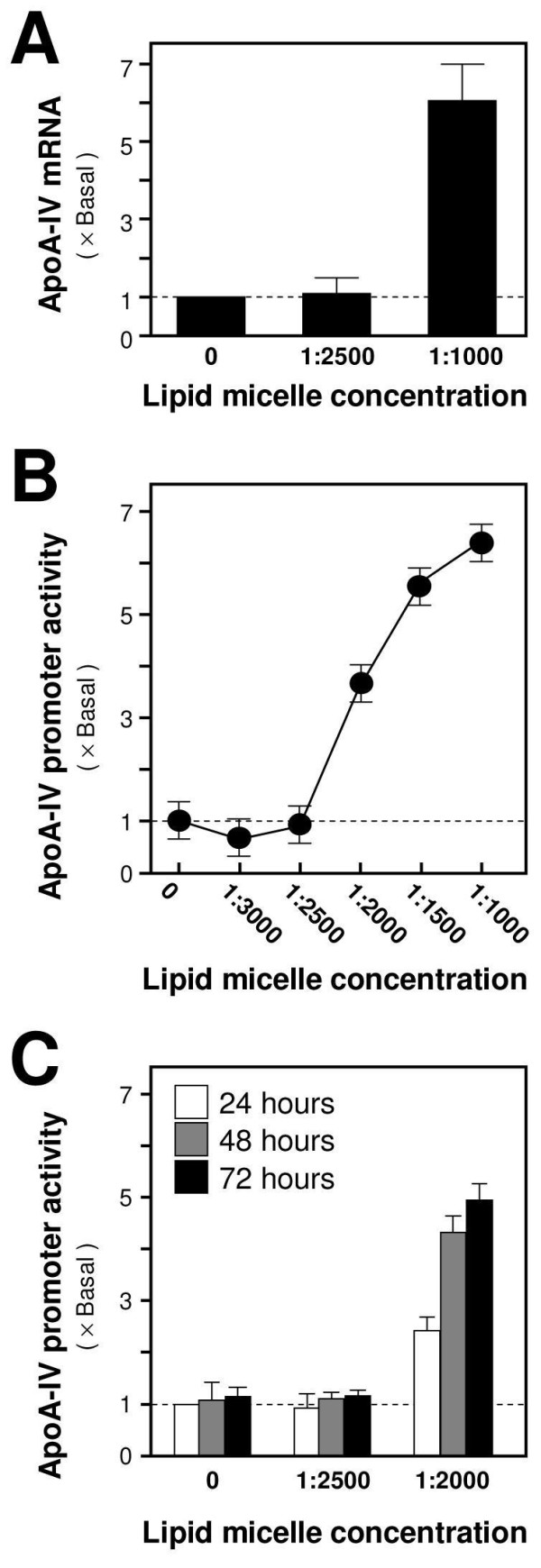
**Transcriptional activation of ApoA-IV mRNA by lipid micelles**. **A**: Caco-2/TC7 cells were treated with lipid micelles (1:1000 and 1:2500) for 24 hours. Total RNAs were extracted and reverse transcribed to cDNA for real-time PCR analysis. The mRNA levels of ApoA-IV were determined by the Ct-value method and normalized by mRNA level of a house keeping gene 18S rRNA. **B**: Caco-2/TC7 cells were treated with various concentrations of lipid micelles for 48 hours. Cultures were collected for luciferase assay to determine ApoA-IV transcription. **C**: Caco-2/TC7 cells were treated with lipid micelles (1:2500 and 1:2000) for 24, 48 and 72 hours to measure the time-dependent regulation of ApoA-IV. Data are expressed as mean ± SD of the multiple of Basal (i.e. buffer-treated control set as one) and number of independent experiments (*n*) = 5.

ApoA-IV was first chosen for the investigation of anti-obesity effect due to its potential role in modulating food intake [17-19]. Accordingly, a promoter-reporter system containing a human ApoA-IV promoter (about 230 bp) tagged with a luciferase reporter gene was employed [27]. This reporter construct was stably transfected into cultured Caco-2/TC7 cells for the screening of potential drugs that regulate the transcriptional activity of ApoA-IV promoter in gut cells. The functionality of this reporter construct was validated by its responsiveness to high concentration of lipid. Cultured Caco-2/TC7 cells were treated with lipid micelle (an artificial mixture of lipids mimicking the duodenal micelles after ingestion) at concentrations of 1:1000 and 1:2500. After a 24-hour treatment, total RNAs were extracted to quantify the amount of ApoA-IV mRNA by a quantitative PCR. Results showed that the expression of ApoA-IV mRNA was not changed by the lipid micelle at the concentration of 1:2500, possibly due to the insufficient amount of lipid micelle to stimulate gene transcription (Figure 1A). However, the induction effect was observed at a higher concentration of 1:1000; the ApoA-IV mRNA was up-regulated to nearly 6 folds compared with the buffer-treated control (Figure 1A). These results confirmed the previous findings that high-fat diet increases the ApoA-IV expression in gut cells [30].

To assess the transcriptional activity of the ApoA-IV promoter, we treated Caco-2/TC7 cells with various concentrations of lipid micelles (1:1000 to 1:3000) for 48 hours and then collected them for luciferase activity. The addition of lipid micelles increased the promoter's activity in a dose-dependent manner. Induction of over six folds was observed in the Caco-2/TC7 cells treated with 1:1000 lipid micelles (Figure 1B). The concentrations of lipid micelles from 1:2000 to 1:1000 were effective in activating the promoter, which was consistent with the findings that ApoA-IV mRNA expression with a concentration at 1:2500 did not produce any response (Figure 1A and Figure 1B). Finally, the optimal treatment time was determined for inducing ApoA-IV promoter activity by lipid micelles. Cultures were treated with two concentrations of lipid micelles (1:2500 and 1:2000) at various time points (i.e. 24, 48 and 72 hours). The promoter activity induced by lipid micelles at 48 hours was similar to that at 72 hours, suggesting that treatment time of 48 hours was sufficient for activation (Figure 1C). Activation was not observed at 1:2500. Therefore, the ApoA-IV promoter is a suitable screening tool in Caco-2/TC7 enterocytes.

### Effects of Chinese medicinal herbs on ApoA-IV transcription

**Figure 2:**
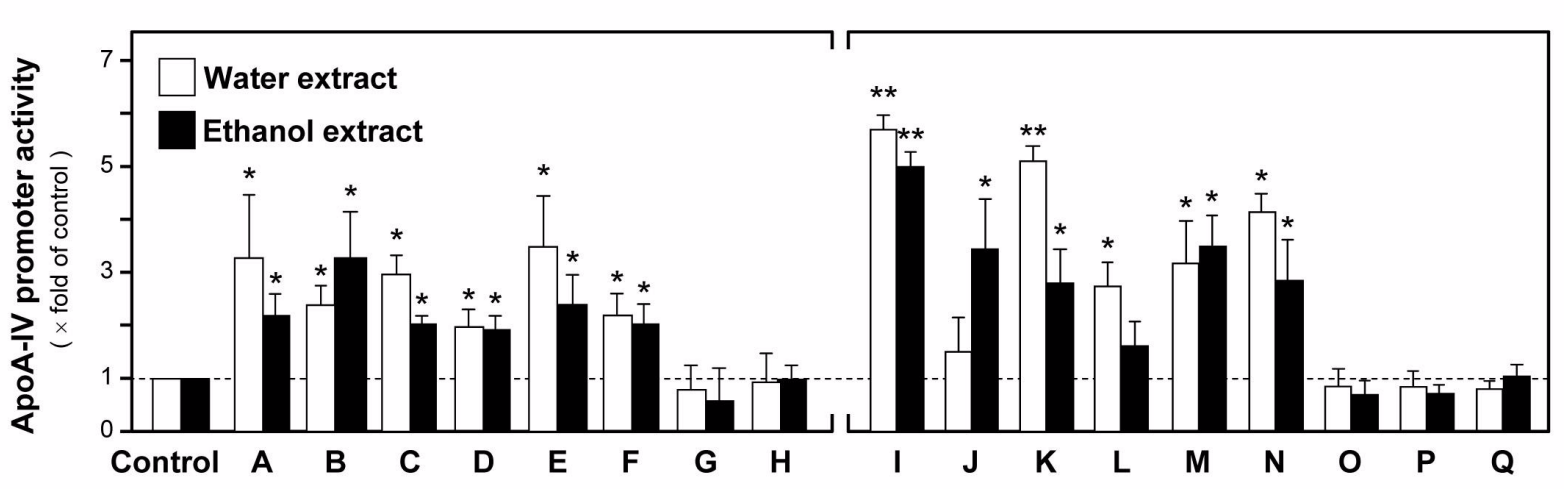
**Transcriptional activation of ApoA-IV by Chinese medicinal herbs**. Caco-2/TC7 cells were treated in various groups with water (1 mg/ml) or ethanol (0.1 mg/ml) extracts of the Chinese medicinal herbs for 48 hours. Luciferase activity was measured. Left: **A**: *Rhizoma Alismatis*; **B**: *Fructus Crataegi*; **C**: *Semen Coicis*;**D**: *Rhizoma Atractylodis Macroczphalae*; **E**: *Rhizoma Atractylodis*; **F**: *Sclerotium Poriae Cocos*; **G**: *Semen Cassiae*; **H**: *Folium Sennae*); Right: **I**: *Radix Angelica Sinensis*; **J**: *Rhizoma Curcumae*; **K**: *Flos Chrysanthemi*; **L**: *Radix et Rhizoma Notoginseng*; **M**: *Folium Nelumbinis*; **N**: *Herba Taraxaci*; **O**: *Pericarpium Citri Retiiculatae*; **P**: *Fructus Schisandrae*; **Q**: *Frutus Mori*. Data are expressed as mean ± SD of the multiple fold of control (i.e. buffer-treated control set as one) and number of independent experiments (*n*) = 5; *P *< 0.05 (*); *P *< 0.01 (**).

Water and ethanol extracts of 17 Chinese medicinal herbs in water and ethanol extractions were screened for their effect in regulating ApoA-IV promoter activity in cultured Caco-2/TC7. The herbs were divided into two groups, namely those that are used to treat obesity (A-H) and those that are not used to treat obesity (I-Q). The powders of water and ethanol extracts of these herbs were dissolved in water and DMSO respectively. The pH value of each solution in the medium was measured to ensure that the cell culture condition was not affected by the addition of the herb itself. The results showed that more than half of the herbs in both groups induced the ApoA-IV promoter activity (Figure 2). In the anti-obesity herb group, *Rhizoma Alismatis *(A), *Fructus Crataegi *(B), *Semen Coicis *(C), *Rhizoma Atractylodis Macrocephalae *(D) and *Rhizoma Atractylodis *(E) increased the ApoA-IV promoter activity by more than two folds (Figure 2). Moreover, both water and ethanol extracts of the herbs demonstrated similar effects, suggesting high availability of active ingredients in the herbs. In the group of herbs not documented for anti-obesity treatment, *Radix Angelica Sinensis *(I), *Rhizoma Curcumae *(J), *Flos Chrysanthemi *(K), *Radix Notoginseng *(L), *Folium Nelumbinis *(M) and *Herba Taraxaci *(N) significantly up-regulated the transcriptional activity of ApoA-IV promoter after 48 hours of treatment (Figure 2).

**Figure 3:**
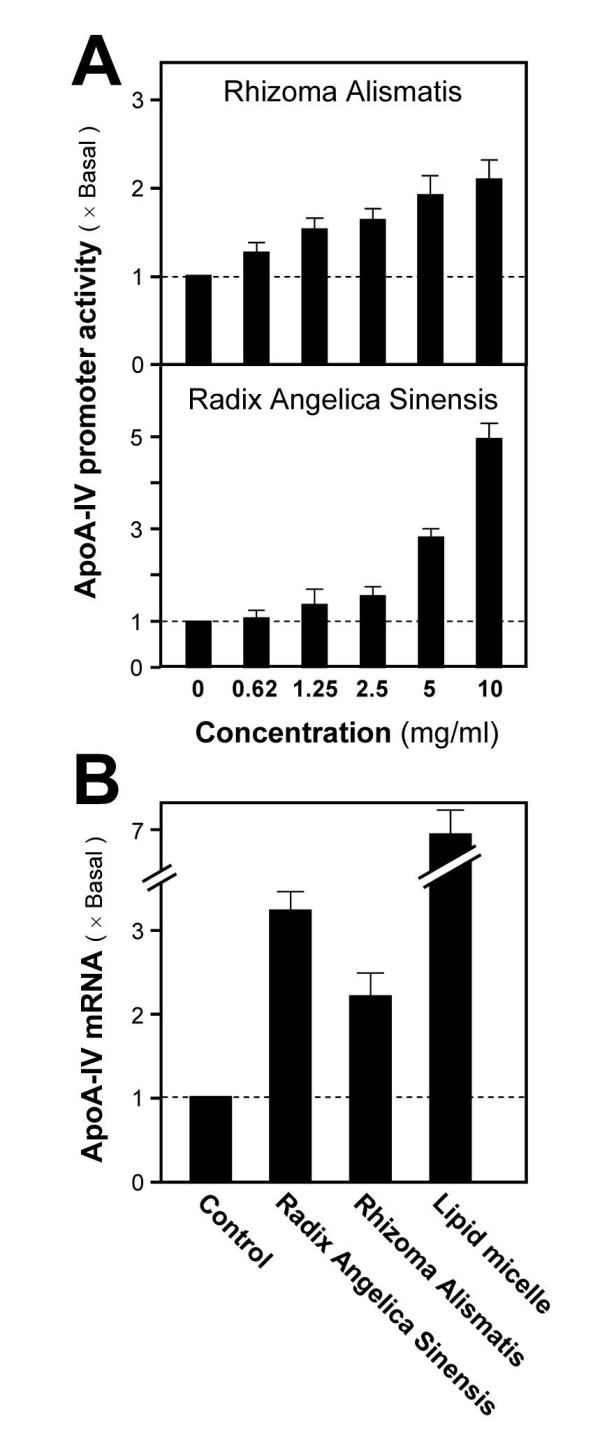
**Stimulation of ApoA-IV mRNA by *Rhizoma Alismatis *and *Radix Angelica Sinensis***. **A**: Caco-2/TC7 cells were treated with water extracts (0–10 mg/ml) of *Rhizoma Alismatis *and *Radix Angelica Sinensis *for 48 hours. Luciferase activity was measured. **B**: Caco-2/TC7 cells were treated with *Rhizoma Alismatis *and *Radix Angelica Sinensis *(1 mg/ml) for 24 hours. The change of ApoA-IV mRNA was determined by RT-PCR analysis. Data are expressed as mean ± SD of the multiple of Basal (i.e. water-treated control set as one) and number of independent experiments (*n*) = 5.

Water extracts of *Rhizoma Alismatis *(A) and *Radix Angelica Sinensis *(I) were further examined for their dose-dependent effect in Caco-2/TC7. After 48 hours of treatment at various concentrations (0 to 10 mg/ml), the luciferase activity was stimulated gradually in response to the increase concentrations of extracts (Figure 3A). Lastly, the same activation effect of *Rhizoma Alismatis *and *Radix Angelica Sinensis *in up-regulating the ApoA-IV mRNA expression was revealed in treated Caco-2/TC7 cells (Figure 3B). Lipid micelles served as the positive control for mRNA analysis. These results showed that *Rhizoma Alismatis *and *Radix Angelica Sinensis *stimulated the ApoA-IV promoter activity.

### Inhibition of lipogenesis in differentiated 3T3-L1 adipocytes

**Figure 4:**
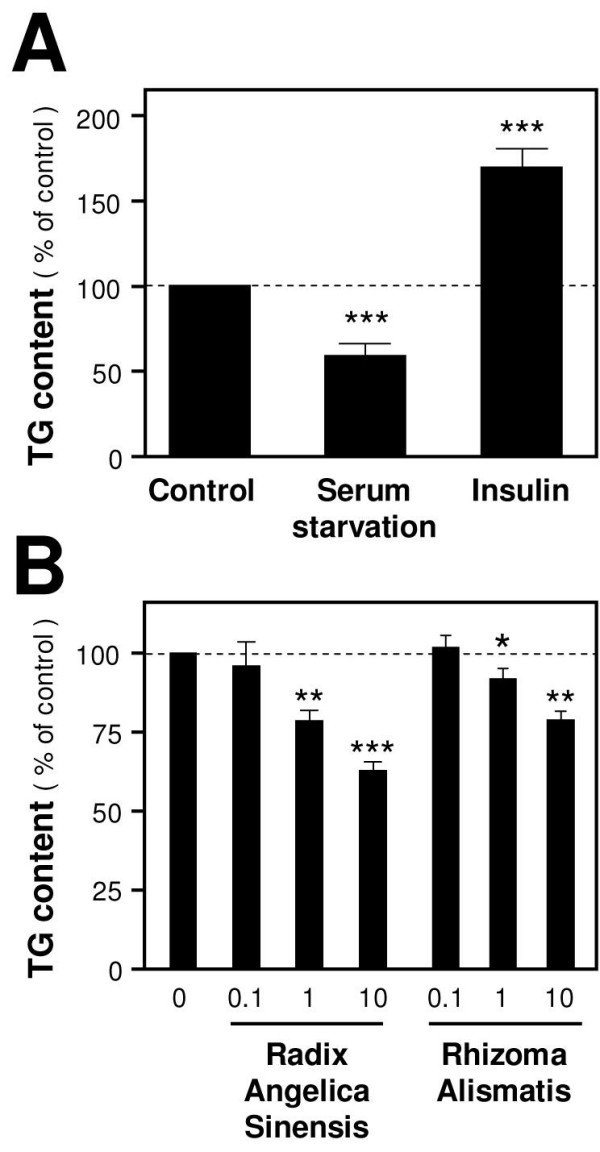
**Reduction of TG content by *Radix Angelica Sinensis *and *Rhizoma Alismatis *in differentiated 3T3-L1 adipocytes**. **A**: Lipogenic differentiated 3T3-L1 cells were either serum-starved or treated with insulin (10 μg/ml) for three days, and then stained by Oil red O dye. The amount of stained TG (red color) was quantified at 490 nm absorbance. **B**: Lipogenic differentiated 3T3-L1 cells were treated with *Rhizoma Alismatis *and *Radix Angelica Sinensis *(0.1, 1 and 10 mg/ml) for 72 hours. The reduction of TG content was measured. Data are expressed as mean ± SD of the percentage of control (i.e. water-treated control set as 100) and number of independent experiments (*n*) = 5; *P *< 0.01 (**); *P *< 0.001 (***).

The 3T3-L1 pre-adipocyte model for adipogenesis studies [28,33,34] was employed to further determine the anti-obesity activity of *Rhizoma Alismatis *and *Radix Angelica Sinensis*. Induced by a chemical cocktail, the pre-adipocytes were differentiated, indicated by morphological changes and accumulation of triglyceride (TG). The TG vesicles inside the cells were stained by Oil Red O dye for visualization and quantification. The differentiated 3T3-L1 cells were serum-starved or treated with insulin for three days to confirm that TG formation did respond to changes. The TG content decreased about 50% in the serum starvation group, and increased to 160% in the insulin group (Figure 4A). The differentiated 3T3-L1 cells were treated with various concentrations of *Rhizoma Alismatis *and *Radix Angelica Sinensis *for three days. With the addition of 10 mg/ml and 1 mg/ml water-extracts, both *Rhizoma Alismatis *and *Radix Angelica Sinensis *reduced the TG levels to varying extents (Figure 4B); the most significant effect (over 30% TG reduction) was observed in *Radix Angelica Sinensis *treatment at 10 mg/ml. These results showed that both *Rhizoma Alismatis *and *Radix Angelica Sinensis *inhibited the formation of TG.

## Discussion

The potential of some Chinese medicinal herbs against obesity in terms of stimulating ApoA-IV promoter activity in gut cells and reducing TG content in adipocytes was tested in the present study. *Rhizoma Alismatis *(A), *Fructus Crataegi *(B), *Semen Coicis *(C), *Rhizoma Atractylodis Macroczphalae *(D), *Rhizoma Atractylodis *(E) and *Sclerotium Poriae Cocos *(F), the herbs tradtionally used to treat obesity, were shown to activate ApoA-IV promoter activity in Caco-2/TC7 cells. In addition, the extract of *Fructus Crataegi *(B) in hyperlipidemia mice displayed the lipid regulating function [35]. The dehydrotrametenolic acid isolated from *Sclerotium Poriae Cocos *(F) promotes the differentiation of adipocyte *in vitro *and acts as an insulin sensitizer *in vivo *[36]. *Rhizoma Alismatis *(A) was shown to have *in vitro *anti-diabetic effect [37] and it is involved in an herbal formulation for lowering plasma glucose [38]. These findings, together with our data in stimulating ApoA-IV promoter, were in agreement with the traditional prescription of those TCMs for anti-obesity activity. In contrast, *Semen Cassiae *(F) and *Folium Sennae *(H) did not exert any stimulatory effect on promoter activity here. A possible explanation would be that single herb might not be effective in targeting obesity. The promising biological effect would be obtainable only in the presence of other appropriate herbs in a decoction mixture. The uniqueness of a precise combination of different herbs is demonstrated in a traditional decoction *Danggui Buxue Tang*; the chemical compositions and biological efficacies significantly controlled by *Radix Astragali *and *Radix Angelica Sinensis *at a 5:1 ratio [39-42].

It is worth noting that some herbs from the Chinese medicinal herbs not traditionally used to treat obesity (I-Q), such as *Radix Angelica Sinensis *(I) and *Radix Notoginseng *(L) induced ApoA-IV transcription. *Radix Angelica Sinensis *is traditionally used to treat menstrual disorders [43,44] and modulate the immune system [45]. *Radix Notoginseng *is used to promote blood circulation, remove blood stasis, induce blood clotting, relieve swelling and alleviate pain [46-48]. The present study shows that *Radix Angelica Sinensis *(I), *Rhizoma Curcumae *(J), *Flos Chrysanthemi *(K), *Radix Notoginseng *(L), *Folium Nelumbinis *(M) and *Herba Taraxaci *(N) increase ApoA-IV transcription and may also be used to treat obesity.

Adipocytes are in the adipose tissue where triacylglycerol is stored as a fuel for the body. Excess adipose tissue can lead to insulin resistance, thereby increasing the risk of type II diabetes and cardiovascular diseases [49]. Adipogenesis of 3T3-L1 pre-adipocyte cells is often used in anti-obesity studies. The mature adipocytes have cytoplasmic lipid vesicles containing newly synthesized TG [49]. In the present study, we found that both *Rhizoma Alistmatis *(A) and *Radix Angelica Sinensis *(I) effectively decreased fat accumulation in 3T3-L1 adipocytes in a dose-dependent manner; *Radix Angelica Sinensis *treatment reduced TG content up to 40% at a dose of 10 mg/ml. These findings suggest that *Rhizoma Alismatis *and *Radix Angelica Sinensis *may possess multi-functional activities against obesity.

## Conclusion

The present study suggests that *Rhizoma Alistmatis *and *Radix Angelica Sinensis *may be potentially useful in treating obesity as they stimulate ApoA-IV transcription and reduce TG formation.

## Abbreviations

ApoA-IV: apolipoprotein A-IV; Ct: cycle threshold; SD: standard deviation; TG: triglyceride.

## Competing interests

The authors declare that they have no competing interests.

## Authors' contributions

AG carried out the experiments and drafted the manuscript. RC contributed to the study design and manuscript revision. AC, JL and IC assisted in performing the experiments. TD and KT contributed to the study design. BL supervised the study. All authors read and approved the final version of the manuscript.

## Supplementary Material

Additional file 1 **The table provides the pharmaceutical names, pinyin names, voucher specimen numbers and characterization of the Chinese medicinal herbs in the present study.** Click here for file
