# Supplementary material for: Stimulation of Apolipoprotein A-IV expression in Caco-2/TC7 enterocytes and reduction of triglyceride formation in 3T3-L1 adipocytes by potential anti-obesity Chinese herbal medicines
Source: Chin Med. 2009 Mar 26;4:5. doi: 10.1186/1749-8546-4-5 (PMC2676280; doi:10.1186/1749-8546-4-5)
Supplement: Additional file 1 — The table provides the pharmaceutical names, pinyin names, voucher specimen numbers and characterization of the Chinese medicinal herbs in the present study. [file 1749-8546-4-5-S1.doc]

**Voucher specimen numbers and characterization of the Chinese medicinal herbs in this study**

| **Sample code** | **Pharmaceutical name** | **Name in *Pinyin*** | **Voucher number** | **Organoleptic characteristics** |
| --- | --- | --- | --- | --- |
| A | *Rhizoma Alismatis* | *Zexie* | 2007-230R | Subpherical, elliptical or ovate, 2-7cm long, 2-6cm in diameter. Externally yellowish-white or yellowish-brown, with irregular transverse-annular shallow furrows and numerous small raised fibrous root scars, occasionally tuberculate bud scars attached to the base. Texture compact, fracture yellowish-white, starchy, with numerous small pores. Odor, slight; taste, slightly bitter. |
| B | *Frutus Crataegi* | *Shanzha* | 2008-731O | Rounded slices shrunken and uneven, 1-2.5cm in diameter, 2-4mm thick. Externally red, wrinkled, with small grayish-white spots. Pulp dark yellow to pale brown. Transverse slices of the middle part showing 5 pale yellow kerns, mostly fallen off, and loculi hollowed. Some slices exhibiting a slender fruit stalk or remains of calyx. Odor, slightly aromatic; taste, sour and slightly sweet. |
| C | *Semen Coicis* | *Yiyiren* | 2008-455O | Broad ovoid or elongated-elliptical, 4-8mm long, 3-6mm wide. Externally milky white, smooth, occasionally with yellowish-brown testa; One end obtusely rounded, the other end relatively broad and slightly dented with 1 pale brown dotted hilum; Dorsal surface rounded and protruding; ventral surface having 1 relatively broad and deep longitudinal furrow. Texture hard, fracture white and starchy. Odor, slight; taste, slightly sweet. |
| D | *Rhizoma Atractylodis Macrocephalae* | *Baizhu* | 2007-233R | Irregular plump masses, 3-13cm long, 1.5-7cm in diameter. Externally grayish-yellow or grayish-brown, with warty protruding, interrupted longitudinal wrinkles and grooves, and scars of fibrous rootlets; remains of stems and bud scars attached to the apex. Texture hard, uneasily broken, fracture uneven, yellowish-white to brownish, scattered with brownish-yellow dotted oil cavities. The baking-dried material horny and relatively dark colored or craked in section view. Odor, aromatic; taste, sweet and slightly pungent, viscous on chewing. |
| E | *Rhizoma Atractylodis* | *Cangzhu* | 2007-232R | Irregularly moniliform or nodular-cylindrical, somewhat curved, occasionally branched, 3-10cm long, 1-2cm in diameter. Externally grayish-brown, wrinkled, transversely twisted-lined, with remains of rootless, and stem scars or remains of stems attached at apex. Texture compact, fracture yellowish-white or grayish-white, scatted with many orange-yellow or brownish-red oil cavities and crystallized out as white fine needle crystals after exposing for a long time. Odor, characteristic; taste, sweetish, pungent and bitter. |
| F | *Sclerotium Poriae Cocos* | *Fuling* | 2008-733O | Subglobose, ellipsoid, oblate or irregular-shaped, variable in size. The outer skin thin and rough, brown to blackish-brown, conspicuously shriveled and striated. Texture hard and compact, fracture granular, some cracked, the outer layer pale brown, inner part white, rarely reddish, some showing the penetrating roots of pine in the centre. Odor, slight; taste, weak and sticky when chewed. |
| G | *Semen Cassiae* | *Juemingzi* | 2008-103O | Slightly rhomboidal-cuboid or shortly cylindrical, both ends parallel and oblique, 3-7mm long, 2-4mm wide. Externally greenish-brown or dark brown, smooth and lustrous. One end relatively even, the other end oblique and acuminate, dorsal and ventral surfaces exhibiting a raised rib respectively, with an obliquely symmetrical and paler-colored dented line on each side of a rib. Texture hard and uneasily broken. Testa thin, cotyledons 2, yellow, S-shaped. Odor, slight; taste, slightly bitter. |
| H | *Foilum Sennae* | *Fanxieye* | 2008-2190 | Elongated ovate or ovate-lanceolate, 1.5-5cm long, 0.4-2cm wide, margin entire, apex acute, base slightly asymmetrical. Upper surface yellowish-green, lower surface pale yellowish-green, glabrous or nearly glabrous, veins slightly prominent. Texture leathery. Odor, slight and characteristic; taste, slightly bitter and mucilaginous. |
| I | *Radix Angelica Sinensis* | *Danggui* | 2007-198R | Somewhat cylindrical, 3-5 or more branched at the lower part, 15-25cm long. Externally yellowish-brown to brown, longitudinally wrinkled and transversely lenticel-like protruded. Root stocks (*Guitou*) 1.5-4cm in diameter, annulated, apex obtuse and rounded, showing purple or yellowish-green remains of stems and leaf sheaths; main roots (*Guishen*) lumpy on the surface; branching roots (*Guiwei*) 0.3-1cm in diameter, the upper portion thick and the lower portion thin, mostly twisted and exhibiting a few rootlet scars. Texture flexible, fracture yellowish-white or yellowish-brown, bark thick, showing some clefts and numerous brown dotted secretary cavities, wood paler in color, cambium ring yellowish-brown. Odor, strongly aromatic; taste, sweet, pungent and slightly bitter. |
| J | *Rhizoma Curumae* | *Ezhu* | 2007-240R | Ovoid, elongate ovoid, conical or elongate fusiform, frequently apex obtuse, base obtuse and rounded, 2-8cm long, 1.5-4cm in diameter. Externally grayish-yellow to grayish-brown, the upper part conspicuously raised-annulated and with rounded and slightly dented rootlet scars, or remaining rootlets, some exhibiting a row of concave bud scars and subrounded lateral rhizome scars on two sides, respectively, and some showing knife cut traces. Texture heavy and hard, fracture grayish-brown to bluish-brown, waxy, usually attached with grayish-brown power, bark and stele easily detachable, endodermic ring deep brown. Odor, slight aromatic; taste, slight bitter and pungent. |
| K | *Flos Chrysanthemi* | *Juhua* | 2008-473O | Obconical or cylindrical, sometimes slightly compressed to fan-shape, 1.5-3cm in diameter, separated. Involucre dish-shaped; bracts 3-4 layers, ovate or elliptical, herbaceous, yellowish-green or brownish-green, the outer surface covered with white hairs, margin membranous. Receptacle hemispherical, without paleas or palea hairs. Ligulate florets several rows, female, marginal, whitish, strongly straight and upward, longitudinally folded and wrinkled. Golden glandular spots scattered; tubular florets abundant, hermaphrodite, occurring in the centre, concealed by the ligulate florets, yellow, with 5 terminal teeth. Achenes undeveloped, pappus absent. Texture light and soft, lax and brittle when dried. Odor, pleasant and aromatic; taste, sweet, slightly bitter. |
| L | *Radix Notoginseng* | *Sanqi* | 2007-197R | Main roots subconical or cylindrical, 1~6cm long, 1~4cm in diameter. Externally grayish-brown or grayish-yellow with interrupted longitudinal wrinkles and branch root scars. Stem scars at the apex surrounded by warty protruding. Texture heavy and compact, fracture grayish-green, yellowish-green or grayish-white, wood slightly radially arranged. Odor, slight; taste, bitter but afterwards sweetish. |
| M | *Folium Nelumbinis* | *Heye* | 2008-113O | Semicircular or plicate, subrounded when spread, 20-50cm in diameter, margin entire or slightly sinuous. Upper surface dark green or yellowish-green, relatively rough, lower surface pale grayish-brown, relatively smooth, with 21-22 thick veins, radiating from the centre to the border, with convex remains of petiole in the centre. Texture fragile, easily broken. Odor, slightly aromatic; taste, slightly bitter. |
| N | *Herba Taraxaci* | *Pugongying* | 2007-97O | Crumpled and rolled masses. Roots conical, frequently curved, 3-7cm long; externally brown, crumpled; the root stock with brown or yellowish-white hairs, some fallen off. Leaves basal, frequently crumpled and broken, when whole, oblanceolate, greenish-brown or dark grey; apex acute or obtuse; margin lobate or pinnatifid; base becoming narrow downwards to petioleshape; midrib of lower surface distinct. Pedicels 1 or more, each with a capitulum on the top; involucre several layers, the inner layer relatively long; corolla yellowish-brown or pale yellowish-white. Some showing numerous long-elliptical achenes with white aigrets. Odor, slight; taste, slightly bitter. |
| O | *Pericarpium Citri Reticulatae* | *Chenpi* | 2008-814O | Often peeled in several lobes connecting at the base, or in irregular slices, 1-4mm thick. Outer surface orange-red to reddish-brown, with fine wrinkles and sunken oil cavity spots; inner surface pale yellowish-white, rough, bearing yellowish-white or yellowish-brown vein-like vascular bundles. Texture slightly hard and fragile. Odor, aromatic; taste, pungent and bitter. |
| P | *Fructus Schisandrae Chinensis* | *Wuweizi* | 2007-175O | Irregularly spheroidal or compressed-spheroidal, 5-8 mm in diameter. Externally red, purplish-red or dull red, shrunken, oily, with soft pulp, sometimes externally blackish-red or covered with “white frost”. Seeds 1-2, reniform, externally brownish-yellow, lustrous, testa thin and fragile. Odor of pulp, slight; taste, sour. Odor of seeds, aromatic on crushing; taste pungent and slightly bitter. |
| Q | *Fructus Mori* | *Sangshen* | 2008-169O | Collective fruit, aggregated by many small achenes, fruit-spike oblong, 1-2cm long, 5-8mm in diameter. Yellowish-brown, brownish-red to dark purple, with a short fruit stalk. Achene ovoid slightly flattened, about 2mm long and 1mm wide, with 4 fleshy perianthsegments. Odor, slight; taste, slightly sour and sweet. |
